# Update on Adverse Effects of HIV Integrase Inhibitors

**DOI:** 10.1007/s40506-019-00203-7

**Published:** 2019-11-16

**Authors:** Agnieszka Kolakowska, Anaenza Freire Maresca, Intira Jeannie Collins, Johann Cailhol

**Affiliations:** 1 Infectious and Tropical Diseases Department, Avicenne University Hospital, Bobigny, France; 2 MRC Clinical Trials Unit at UCL, Institute of Clinical Trials & Methodology, 90 High Holborn, 2nd Floor, London, WC1V 6LJ UK; 3 Infectious and Tropical Diseases Department, Avicenne University Hospital and Paris 13 University, Bobigny, France

**Keywords:** HIV integrase inhibitors, Drug-related side effects and adverse reactions, Raltegravir, Elvitegravir, Dolutegravir, Bictegravir, Cabotegravir

## Abstract

**Purpose of review:**

The goal of this paper is to provide an up-to-date review of adverse events related to the class of integrase strand transfer inhibitors (INSTIs), which became the class of choice in few years. We sought answers specifically to issues pertaining to neuropsychiatric adverse events, as well as weight gain, which were the two most important categories of adverse events raised in recent studies based on real-life experience. The primary focus of this paper is on adults with a brief summary on pregnant women and children/adolescents.

**Recent findings:**

Dolutegravir (DTG) bears the heaviest burden of neuropsychiatric side effects. Weight gain was reported with all INSTIs, although there are methodological caveats in the analyses and the findings need to be interpreted with caution.

Moreover, due to recent findings on neural tube defects in infants exposed to dolutegravir during their peri-conception period, its use is not recommended for women of childbearing age without proper birth control method, while raltegravir remains the only drug which may be prescribed without caution. Given the importance of cognitive and metabolic co-morbidities in people living with HIV in regard to their quality of life, future research needs to focus on long-term effects of INSTIs in relation to these adverse events. Pharmacogenetics seems to be a promising tool. Safety during pregnancy is also another important issue to further clarify.

**Summary:**

INSTIs are a generally well-tolerated class of antiretrovirals (ARV), and has a higher antiviral potency compared to other classes of ARV.

Clinicians and patients need however to be aware of some red flags when starting with and monitoring patients on INSTIs.

All INSTIs can lead to mild increases in creatinine levels, usually without clinical significance, but caution is needed in patients with low eGFR (<30ml/min), when using other nephrotoxic drugs, such as as tenofovir disoproxil.

Neuro-psychiatric (NP) effects are to be monitored with INSTIs, especially with DTG (though reports are at times contradictory); clinicians might want to avoid DTG for patients with history of severe NP symptoms, until clarity is provided.

Weight gain was reported with all INSTIs, especially with DTG, with possible differential effects according to sex and ethnicity (female and non-white patients being at increased risk). This is worrying since patients from African descent are at higher risk of cardio-vascular events and increased body mass index (BMI) can cause further increase metabolic risk. There is possibly an additional effect of tenofovir alafenamide (TAF) on weight increase.

Discrepancies between clinical trials – with low rates of adverse events – and reports from real-life settings might be due partly to under-representation of some groups of patients in clinical trials, and/or the short duration of follow-up, since some adverse effects may only occur after prolonged exposure.

Preliminary data on safety of bictegravir (BIC), from clinical trials and non-trial settings, are very reassuring and seem to show lower rates of adverse events compared to DTG.

Elvitegravir/cobicistat (EVG/cobi) need to be used with caution in patients with other co-morbidities given potential for polypharmacy, as it is the case for aging patients, because of the high potential of drug-drug interactions due to effects of the cobicistat booster.

We are awaiting the release of cabotegravir (CAB), which could represent a good option for patients struggling with adherence, despite injection site reactions.

Pharmacogenetics is a promising way to explore adverse effects occurrence in the INSTI class.

## Introduction

Over the past 40 years, remarkable advance in antiretroviral therapy (ART) has enabled people living with HIV (PLWH) to enjoy longer life expectancy and improved quality of life. Increasing options in drugs constituting the ART regimen allow a more personalized approach to choice of treatments, taking into account tolerability and risk of adverse events. Integrase strand transfer inhibitors (INSTIs), which block the integration of the viral genome into the host genome, are presently one of the most popular drugs used in first-line combination therapy, due to their high potency, good tolerability, low toxicity, and high genetic barrier to resistance, particularly the second-generation INSTIs [1–3].

Raltegravir (RAL) and elvitegravir (EVG) were the first INSTIs to be approved in 2007 and 2012, respectively [4, 5]. However, the emergence of resistance to RAL and EVG (thereby cross-resistance to each other) has been a challenge [1, 6], and subsequently second-generation INSTIs were developed: dolutegravir (DTG) was approved in 2013; bictegravir (BIC), formerly GS-9883, approved in the USA in February 2018; and cabotegravir (CAB), also known as GSK744, now in phase III of clinical development with an anticipated release in 2019 [7–9]. CAB, a structural analog of DTG, displays unique physicochemical and pharmacokinetic properties allowing the formulation to be used as a single oral tablet for daily dosing or as a long-acting nanosuspension for monthly to quarterly intramuscular injection.

**Fig. 1:**
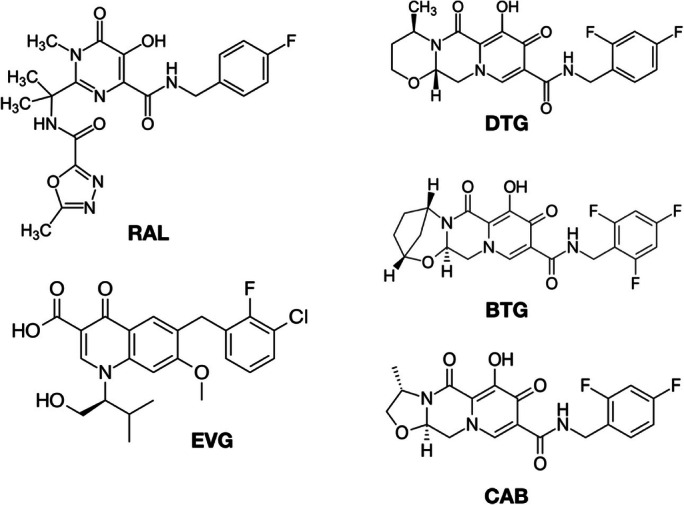
Chemical structures of INSTIs (own creation using Wikipedia license-free images).

INSTIs are generally well tolerated during real-world clinical use. Toxicity profiles for this class of drugs have included neurological, weight gain, and gastrointestinal symptoms [2, 8–10], and in the long-acting CAB, injection-site reactions [10]. Studies have reported INSTIs’ any adverse effect rates (proportions) as 5.7–7.6/100 person-years (3.6–5.8%) for RAL, 3.8–13.9/100 person-years (2.5–13.7%) for DTG [2, 11–13], and 4.4–10.3/100 person-years (3.4–12.3%) for EVG [2, 11–13]. Understanding the differences in adverse drug reaction profile between INSTIs can better inform personalized ART selection (Fig. 1).

## Neuropsychiatric symptoms

Neuropsychiatric (NP) symptoms have been reported with all INSTIs, and their onset is usually described during the first few weeks after introduction. Symptoms include headaches, reduced concentration, anxiety, irritability, dizziness, insomnia, altered dreams, depression, unexplained pain, and more recently, mood changes (see Table 1).

**Table 1:** Characteristics of neuropsychiatric side effects

	RAL %	EVG %	DTG %	Reference
Insomnia	-	-	8	[14]
	30.4	8.7	19	[15••]
	8.5	8.6	7.7	[16]
	-	-	4	[17]
	5	-	6	[18]
	-	3.4	-	[19]
Abnormal dreams	30.4	4.3	9.5	[18]
	-	4.6	-	[19]
Anxiety	-	-	5	[14]
	34.8	17.4	33.3	[15••]
	10.2	6.8	6.6	[16]
	-	-	2	[17]
	6	-	4	[18]
Headache	-	-	17	[14]
	17.4	8.7	33.3	[15••]
	13		14	[18]
	-	5,1	-	[19]
	-	-	3	[17]
Depression	-	-	6	[14]
	14.1	9.5	10.1	[16]
	2	-	3	[20]
	-	-	2	[17]
	5	-	7	[18]
	-	4.3	-	[19]
Suicidality	-	-	2	[14]
	0.2	0.2	0.1	[16]
	1	-	< 1	[18]
NP side effects of any kind	1.7	1.3	2.7	[21]

In clinical trials, there were low and comparable rates of NP symptoms among patients receiving RAL, EVG-COBI, and DTG. However in real-life settings, findings about NP effects have been variable and at times contradictory, although several authors have reported higher rates of NP symptoms than in clinical trials (early or longer term symptoms, leading to discontinuations at times), especially among patients on DTG [21–25]. Reasons for these differences are likely two-tiered. Firstly, assessment of NP symptoms is often difficult, subject to inter-observer variations, and possibly observer bias depending on their awareness of NP effects. Secondly, many scales for the assessment of NP exist, rendering comparisons between studies or between clinical trials and real-life settings challenging especially since the numbers of subjects evaluated in trials are relatively small compared to studies in routine care settings once a drug is approved. The most salient issue for patients is to detect and minimize NP symptoms.

Insomnia is one of the most commonly reported neurologic symptoms associated with INSTI, although studies did not find significant differences between INSTI drugs. Murrell et al. [15••] and Wohl et al. [19] reported quite high occurrences of abnormal dreams, whereas these were rare during clinical trials.

Murrell et al. [15••] did not find any significant difference in rates of anxiety between drugs, but the observed rates were much higher (29.5%) than in clinical trials where they did not exceed 10%.

Headaches are often reported at the beginning of all antiretroviral (ARV) therapies, and they usually gradually disappear.

The proportion reporting headache was substantially higher in Murrell’s study at one-third of patients (33%) on DTG, while other studies reported much lower estimates of 3% [17].

Moreover, some authors found risk factors associated with NP side effects with DTG, with higher risk among women, those aged 60 years and more and those who started abacavir (ABC) at the same time as DTG vs those who did not [11, 23, 26]. Some data on therapeutic drug monitoring suggest that morning dosing or reducing dosage of DTG could reduce the NP effects [26, 27].

Lastly, in a randomized clinical trial (RCT) led by Wohl et al. [28], patients reported more dizziness (6% vs 4%) and more sleep disorders (3% vs 1%) with DTG than with BIC respectively, without information on statistical significance.

Effects of INSTIs on the central nervous system are still being investigated, and new forms of assessments, such as brain integrity measurement, have started to appear [29]. This cross-sectional study showed a reduction of brain volume in patients taking INTSIs, as well as decreased performance in memory and learning, compared to patients not taking INSTIs [29]. Recently, pharmacogenetics analysis showed several single nucleotide polymorphisms associated with some adverse events with INSTIs, paving the way for future research on precision medicine [15••]. Indeed, high inter-individual variations in drug concentrations (and thus risk of adverse events) could be related to inter-individual difference in pharmacokinetics.

## Weight gain

PLWH are at higher risk of cardiovascular disease when compared with the HIV-uninfected population [14, 24]. There is no direct correlation between higher body mass index (BMI) and risk of myocardial infarction in PLWH, but higher BMI is a recognized risk factor for diabetes mellitus, which is a known risk factor for myocardial infarction in the general population and in PLWH [16, 18]. Weight gain and BMI increase are central issues in PLWH, who need to reduce the risk of metabolic disease.

Some reports have highlighted a possible role of DTG in weight gain [20] and of RAL in body fat composition changes [30]. This leads to question the potential class effect of INSTIs in fat gain.

Two RCTs comparing BIC to DTG [28, 31] both reported an increase in body weight ranging from 2.4 to 3.9 kg, for BIC or DTG, at week 96. Information collected through the SCOLTA cohort [24] revealed a significant 1-year BMI increase in patients treated with DTG (*p* = 0.004), RAL (*p* = 0.0004), and EVG (*p* = 0.004). At 1 year, patients in Centers for Disease Control (CDC) stages A and B experienced a mean BMI increase of 0.13 (± 0.06), significantly associated with low baseline BMI (*p* = 0.002) and older age (*p* = 0.0007) at start of treatment. As compared with DRV, RAL patients had a significantly lower BMI modification (*p* = 0.038). Focusing instead on patients in CDC stage C, the mean BMI increase at 1 year was 0.46 (± 0.08) and was associated with lower BMI (*p* = 0.005) and lower CD4+ T cell count (*p* = 0.007) at enrollment. Previous study demonstrated that the greatest increase in BMI occurred during the first year of ARV therapy [32], while SCOLTA cohort demonstrated a significant increase in BMI even in patients who had already been treated for more than 3 years before switching to INSTI-including regimen.

These findings need to be interpreted against the general population data, in which a BMI increase over time was found more likely in people with normal BMI or overweight [33]. In contrast, among PLWH, a higher BMI gain was found in those who had lower baseline BMI values [24]. One of the hypotheses could be that PLWH with lower CD4 count at baseline might have lost weight as a result of various inflammatory marker effects on metabolism before starting the ART (effect of “return to healthy status”).

Studies reporting BMI changes after enrollment of treatment-naïve patients need to consider their baseline weight before disease progression, even if these self-reported data are more subjective. Alternatively, studies on weight gain in treatment experience patients switching to INSTI-based regimens could be easier to interpret.

Norwood et al. looked at BMI changes when patients switched from efavirenz to an INSTI-containing regimen [20]. In their retrospective observational study, the authors found a significant increase in BMI after switching to any INSTI, with the greatest change in DTG-containing regimens. Hill et al. [] pooled data from 4 observational studies (France, Brazil, and 2 in the USA) and reported a significant increase in body weight for patients initiating on or switching to INSTIs. This increase was particularly evident in women (especially non-white) and among patients on an ABC-containing INSTI regimen.

In summary, weight gain seems to be a significant issue for all INSTIs although patients with lipoatrophy could benefit from a switch to INSTIs, as showed in a study by Domingo et al. [35].

## Metabolic disorders

### Glucose metabolism

Abnormally high fibroblast growth factor 21 (FGF21) levels are considered a marker of disturbed metabolism in non-HIV-infected patients with obesity, diabetes, or congenital lipodystrophy [36–38], whereas betaKlotho (KLB) repression is associated with an impairment of glucose uptake and other health effects mediated by FGF21 [39]. Studies in distinct HIV patient cohorts have consistently reported that elevated FGF21 levels are associated with indicators of insulin resistance, insulinemia, and glycemia [38, 40, 41]. Lifestyle interventions in HIV patients that achieve metabolic improvement are also associated with a decline in FGF21 levels that correlates with indications of improved energy metabolism [40].

EVG induces FGF21 and represses KLB, eliciting ER stress/oxidative stress. Other INSTIs are neutral toward the FGF21/KLB system [42•].

Norwood et al. [20] also found a non-significant increase in Hb A1c level when switching from EFV to an INSTI regimen. In contrast, Calza et al. [43] reported reassuring data with over 10-year follow-up, with a significant decrease in homeostasis model assessment (HOMA) of insulin resistance index after a switch from PI/r to INSTIs.

### Lipid metabolism

The SPIRAL study [44], which switched patients from PI/r to RAL-based regimens, reported better lipid profile at 48 weeks and significant improvements in several cardiovascular biomarkers associated with inflammation, insulin resistance, and hypercoagulability resulting in reduction of risk for coronary heart disease. In 2 RCTs comparing BIC vs DTG (with similar or same backbone nucleoside reverse transcriptase inhibitors (NRTIs)), at week 96, larger increases from baseline in total and low-density lipoprotein cholesterol rates were reported for patients on BIC compared to DTG, but the introduction of lipid-modifying drugs was very low (< 5%) [31], and not different between BIC and DTG [28, 31].

## Hepatic outcome

Neither alkaline phosphatase (ALP) nor alanine aminotransferase (ALT) level outside the normal range was reported among patients on INSTIs, which suggests that this class of drugs may not significantly modify transaminases unless other factors are involved [45]. In a study by Tebas et al., DTG, when co-administered with ABC and lamivudine, was shown to increase ALP by 50% from baseline at 144 weeks [46]. Additionally, when switching from an efavirenz to a RAL-based regimen, serum ALP significantly decreased in the RAL group compared to the efavirenz group at 24 weeks []. Similarly, none of the grade 3 and 4 liver abnormalities were related to study drugs BIC and DTG, in 2 RCTs [28, 31].

## Renal outcome

All INSTIs have been associated with an increase in creatinine levels. In vitro data have shown DTG to inhibit organic cation transporter 2 (OCT2) on the basolateral side of proximal tubular cells []. Therefore, DTG can block tubular uptake of creatinine from the blood, leading to increased serum creatinine and decreased eGFR or CrCl, without changing effective GFR [49]. In the SPRING-2 trial, during the first 2 and 3 weeks of treatment, the mean estimated CrCl decreased by 16.5 mL/min in the DTG group compared with 5.4 mL/min in the RAL group at the end of weeks 4 and 5, without proteinuria (ratio proteinuria to creatininuria) in both groups [5]. This change was non-progressive. The VIKING trial [50], which assessed the effectiveness of additional DTG in patients who failed to suppress HIV replication, reported a non-progressive creatinine increase (12.38 mmol/L for both cohorts, 50 mg of DTG once-daily dose and twice-daily regimen), plateauing at 4 weeks after initiation of therapy. The SPRING-2 study showed a small increase in the mean serum creatinine of 8.2 mmol (with a decrease in mean creatinine clearance (CrCl) of 9.3 mL/min) but no evidence of any proteinuria in RAL-treated patients after 48 weeks of therapy [5]. The SAILING study [51] exhibited similar small increases in creatinine at week 2, approximately 4 and 10 mmol/L in the RAL and DTG treatment arms, respectively, that remained stable for the duration of the 48-week trial.

It has been suggested, given the similarities, that RAL may have an effect on tubular function similar to that of DTG. However, in a study of Gupta et al. [], 30 individuals who switched from EFV to RAL presented an increased serum creatinine and cystatin C, as well as a decrease in GFR (measured by cystatin C clearance) by 8.50 mL/min/1.73 m^2^ in the switch group. This finding might suggest a possible genuine reduction in renal function rather than an effect on creatinine by renal tubular cells. Furthermore, unlike DTG, RAL has no effect on OCT2.

There is no difference in renal function between EVG boosted with ritonavir and RAL with a median decrease in eGFR from baseline to 96 weeks of 10.8 mL/min/1.73 m^2^ in the EVG arm compared to 11.7 mL/min/1.73 m^2^ for RAL, and with no documented formal renal adverse effects [52]. As for cobicistat (COBI), it inhibits the tubular secretion of creatinine and a rise in serum creatinine is expected, with no effect on the actual GFR [53].

BIC and DTG seem to lead to similar decrease in eGFR at week 96 in a RCT comparing BIC/FTC/TAF vs DTG/FTC/TAF [31].

In summary, clinicians should monitor closely the renal function for abnormal decrease in eGFR, as compared to an expected decrease of approximately 10 mL/min/1.73 m^2^. Unfortunately, there is currently no alternative way of routinely assessing renal function that would not be affected by INSTI and COBI.

## Rhabdomyolysis

RAL-based therapy is the only INSTI associated with a higher prevalence of symptomatic skeletal muscle toxicity, which does not seem to be concentration or time dependent, nor associated with elevated CK. Proximal myopathy may be an uncommon but authentic side effect of RAL exposure [54].

## Gastrointestinal outcome

Diarrhea was reported in 18% of RAL subjects [51], while the STARTMRK study reported 1.0% [55]. Eighteen percent of patients receiving DTG in the FLAMINGO trial [45] and 20% in the SAILING study [51] experienced diarrhea, whereas this rate was only 5% with DTG/ABC/3TC [17]. The occurrence of diarrhea reported for EVG/FTC/TDF was respectively of 25% and 26% at 96 and 144 weeks [19] and in LATTE-2, 20% in the oral group (CAB/ABC/3TC) and 28% in the monthly injectable group (CAB/RPV) [7]. With an overall occurrence of diarrhea in 29.5% of subjects, RAL, EVG, and DTG grouping yielded 30.4%, 26.1%, and 30.9%, respectively, without association with a particular regimen, in Murrell’s observational study (*p* = 0.955) [15••].

Nausea has been previously reported in 8% of RAL subjects in SAILING study [51] and 3% in the STARTMRK results [55]. In the FLAMINGO study [45], nausea was present in 17% of subjects receiving DTG and 2% in a DTG/ABC/3TC regimen [17]. EVG/FTC/TDF showed 22% (96 weeks) and 23% (144 weeks) occurrence of nausea [19] and in LATTE-2 in 16% in both oral and intramuscular groups [7]. Murrell et al. [15••] reported that nearly one in five subjects (18.2%) experienced nausea. DTG group exhibited slightly higher occurrence at 23.8%, while EVG and RAL tied at 13.0%. No significant difference (*p* = 0.459) was found between regimen and nausea occurrence.

The trial “1475” was 60 weeks of blind induction with BIC/FTC/TAF or DTG/FTC/TAF, followed by a continuation with/switch to BIC/FTC/TAF, with a total of 91 enrolled persons. At week 72, among 30 participants who switched from DTG to BIC, there was only 1 diarrhea and 1 nausea events (grade 1), related to BIC, without treatment discontinuation [9].

Stellbrink et al. [31] reported fewer gastrointestinal adverse effects in patients receiving BIC, compared to DTG, in their 96-week RCT (9% vs 14%, without statistical significance). Wohl et al. [28] also reported significantly fewer gastrointestinal adverse events with BIC compared to DTG, especially for nausea (6% vs 17%, *p* < 0.0001).

## Aging patients

Abnormal platelet count is a predictor of mortality in the elderly [56]. In the SPIRAL trial [44], a switch from PI/r to RAL led to a slight reduction of platelet count over time of follow-up (mean values, from 240,936/mm^3^ to 200,731/mm^3^, *p* = 0.02). The result remained statistically significant when hepatitis B and C co-infected patients were excluded from the analysis [mean values, from 230,300/mm^3^ to 197,125/mm^3^ *p* = 0.04]. Ral-Age study [57•] reported that switching to a RAL-containing regimen in PLWH over 60 years old showed a statistically significant reduction of median values of triglycerides (*p* = 0.0001) as well as of cholesterol at 36 months of follow-up (*p* = 0.0023). Significantly increased dosages of DTG were found in patients > 60 years [26], potentially explaining reported association between age and DTG NP effects.

## Drug–drug interactions

RAL is better absorbed in an environment at higher pH. Combined use of omeprazole and RAL in healthy subjects led to a 212% increase of RAL plasma concentrations [], but it is not expected that concomitant omeprazole use will lead to a lower tolerability of RAL. PLWA often have achlorhydria or hypochlorhydria, and hence the effect of any acid-reducing agent may be less compared to a healthy volunteer.

Dose adjustment is needed only when INSTI is taken with rifampin, where a dose increase of RAL to 800 mg BID is advised, and with unboosted atazanavir, where a dose increase of atazanavir to 600 mg QD or 300 mg BID is recommended [59].

DTG has an excellent profile in terms of drug interactions. Metformin constitutes one of the exceptions, with a significant increase in metformin levels when co-administered with DTG [60]. This should prompt clinicians to closely monitor lactic acidosis symptoms when prescribing both drugs and US Prescribing Information suggests limiting the total daily dose of metformin to 1000 mg.

COBI is a potent inhibitor of cytochrome P450 (CYP) 3A, of which its role is the elimination of several drugs [61], therefore taking pharmokinetic advantage as a booster, to decrease EVG administration. As such, EVG/COBI has important restrictions in terms of co-medications, albeit with lower potency of inhibition than ritonavir [61]. Examples of contraindicated drugs are simvastatin and risks of rhabdomyolysis and the need for dose adjustments with direct oral anticoagulants. Interactions need to be checked upon initiation or switch to these drugs, for instance on the Liverpool website (https://www.hiv-druginteractions.org/). This issue is particularly important in aging patients, who usually have co-morbidities. A study by Demessine et al. showed a significantly higher drug–drug interaction–induced direct and indirect care costs in case of EVG/COBI, compared to other INSTIs [62].

BIC inhibits 2 transporters implied in renal excretion (OCT2 and MATE1). It may thus increase concentrations of drugs eliminated by the same transporters, such as dofetilide, an antiarrhythmic drug. Metformin dosage might also increase with BIC, though of less clinical significance than with DTG [63]. BIC is not recommended to be co-administered with rifampicin or rifabutin, since BIC *C*_through_ might drop significantly [59].

## General symptoms

Murrell et al. [15••] reported in an observational study with long-term follow-up on INSTI, occurrence of fatigue at 33.0% in the whole INSTI group, 34.8% for RAL, 33.3% for DTG, and 30.4% for EVG (*p* = 1.000). In other studies, fatigue was reported in RAL subjects in 1% [55], 7% [51], and 3.9% [45]; in DTG group 6% [45] and 4% [51]; in EVG/FTC/TDF in 13% and 15% of subjects at 96 weeks and 144 weeks, respectively [19].

## INSTIs and pregnancy

Until recently, only 400 mg of BID RAL and DTG was recommended during pregnancy and/or for childbearing age women living with HIV, due to their pharmacokinetic properties and their “clinical relevance.”

The report of increased rate of neural tube defects (NTD) among women exposed to DTG in the periconception period in Botswana (0.94% [64••]; 0.67% Tsepamo Study [65]; vs 0.12% in women not exposed to DTG [64••]) led the World Health Organization (WHO) to recommend avoiding DTG in potential childbearing aged women wishing to become pregnant [66]. However, a modeling study [67] based on data from a randomized trial in Cameroon [68] concluded that DTG + NRTI regime, due to its strong genetic barrier and good tolerability, was the most cost-effective regimen for the prevention of AIDS-related deaths and for the prevention of mother-to-child HIV transmission in low- and medium-income countries, even after taking into account the NTD occurrence [67]. While we wait for updated data from the Botswana Tsepamo study relating to NTD occurrence, access to/use of DTG among women of childbearing age varies widely across countries and the public health policies and guidelines in place.

Given the expected decrease in exposure and/or increased clearance of EVG during pregnancy especially in the second and third trimesters, this drug is not recommended in pregnant women living with HIV [69–71].

Long-acting CAB and its potential decreased exposure would limit its use in pregnant women [69]. BIC *C*_through_ is likely to be reached during the pregnancy, and further prospective evaluations are necessary to prove that pregnancy “does not affect BIC protein binding” [69, ]. Since NTD might be a class effect, caution is needed also with BIC [63].

Peri-conception exposure to RAL is not associated with NTD and therefore remains the only INSTI recommended in high-income countries for women during the peri-conceptional period or during pregnancy (DHHS (USA), the European AIDS Clinical Society [] and Canadian guidelines [74]). Further trials on efficacy and tolerability are needed on the use of RAL QD 1200 mg in this population [69].

## INSTIs in children and adolescents

Data in this group of patients are still scarce, but are expected to increase given the WHO recommendation to position DTG as the preferred first-line regimen for children and adolescents in low- and middle-income countries. IMPACT P1066 is a phase 1/2 open-label, non-randomized multicenter trial, following 122 children living with HIV, aged from 4 weeks to 18 years old for 240 weeks. They all received RAL BID adults’ tablets, chewable tablets, or granules for oral suspension according to their age and weight. One patient had a transient liver function enzyme elevation that was possibly related to treatment and resolved without study’s drug discontinuation. Two participants (1%) had a drug-related allergic rash: one of them was grade 3 at day 7, and had to discontinue the study because of an adverse event [75].

No adverse effect was related to DTG at 48 and 144 weeks in IMPACT P1093 trial, which enrolled 22 treatment experienced adolescents living with HIV [76, 77]. ODYSSEY is a large multi-country trial evaluating the safety and efficacy of DTG-based regimen for the first or second ART as compared to standard of care in 700 children and adolescents. While the main trial is ongoing, the nested PK study has reported no grade 3 or 4 adverse effects in 15 children from Uganda and Zimbabwe receiving 50 mg and 30 mg film-coated DTG in lower weight bands (20–25 kg) [78]. In routine care settings, a French multicenter retrospective study of 50 adolescents beginning DTG between January 2014 and December 2015 described two patients (4%) who experienced NP side effects during follow-up: moderate headache and dizziness, without treatment cessation in one case; and one severe case of dizziness (grade 4), sleeping disorders, and anxiety, resolved with DTG discontinuation []. A National Collaborative UK and Ireland ART children and adolescent study (CHIPS) reported, among 406 children who were introduced INSTI, there were low rates of toxicity/discontinuation and no change in BMI/*z* score on DTG but an increase on RAL [80].

## Injection-site reactions

Injection-site pain was the most frequently reported adverse event of CAB in the LATTE-2 study [7]. Most injection-site reactions were mild or moderate in intensity, with median symptom duration of 3 days. Although injection-site reactions were common, they did not appear to compromise high levels of patient-reported satisfaction, with very few withdrawals resulting from injection-site reactions, two patients (< 1%) through 96 weeks.

## Conclusions

Taken together, studies affirm that all INSTIs are generally well tolerated, although side-effect profiles differ between drugs. Persons who experience adverse effects with one INSTI may tolerate an alternative drug in this class; however, switching from one INSTI to another may result in new side effects.

Comparisons of older and newer drugs can be confounded by shorter follow-up times for newer agents and changing prescribing patterns over time. NRTIs in the backbone regimen might also constitute a confounding factor in analyzing the adverse events.

Rare adverse events may be related to extended use of INSTIs, and long-term follow-up on INSTI are warranted to capture such events.

Future research including precision medicine (i.e., pharmacogenetics) might help in the future in choosing the best INSTI to prescribe for an individual patient.
